# Trans-Anethole Alleviates DSS-Induced Ulcerative Colitis by Remodeling the Intestinal Flora to Regulate Immunity and Bile Acid Metabolism

**DOI:** 10.1155/2023/4188510

**Published:** 2023-09-21

**Authors:** Xu-Hui Li, Li Liu, Wen-Zhong Wu

**Affiliations:** ^1^College of life Science and Technology, Harbin Normal University, Harbin, China; ^2^Department of Gastroenterology, Heilongjiang Red Cross (General Forest Industry) Hospital, Harbin, China; ^3^Department of General Medicine, People's Hospital of Dongfanghong Forestry Bureau, Fuzhou, China; ^4^Department of Pediatrics, Heilongjiang Red Cross (General Forest Industry) Hospital, Harbin, China

## Abstract

Ulcerative colitis (UC) is the most common inflammatory bowel disease (IBD); it is incurable, and the treatment is expensive. Trans-anethole (TA), the main component of fennel, exhibits various biological activities. An increasing number of studies have demonstrated the efficacy of herbal active ingredients in the treatment of UC. This study aimed to investigate the effect and mechanism of TA in UC. In this study, we have experimented on mice with dextran sulfate sodium salt (DSS)-induced UC. The TA group was gavaged with 62.5 mg/kg TA by gavage once daily on days 8–14. To observe the effect of TA on the colon tissue, various investigations were performed, including western blot and immunohistochemistry for intestinal barrier protein expression, TUNEL staining for apoptosis, western blot, and ELISA for inflammation level, flow cytometry for Th17/Treg, LC–MS for blood bile acid content, GC–MS for blood fatty acid content, and 16s RNA for intestinal contents. TA alleviated weight loss in mice with UC; increased colon length; alleviated intestinal mucosal damage; upregulated claudin-1, occludin, and ZO-1 protein expression levels; reduced inflammatory factors in the colon and serum; and alleviated apoptosis. TA reduced fatty acid and bile acid levels by inhibiting colony abundance and reducing Th17/Treg cell differentiation in the colon. We found that TA alleviates DSS-induced UC by remodeling the intestinal flora to regulate immunity and bile acid metabolism.

## 1. Introduction

Colitis is an inflammatory lesion of the inner layer of the colonic mucosa; there are several types of colitis [1]. The pathological process of colitis is unclear, and its pathogenesis involves genetic and immune-mediated causes, infectious agents, drugs, and bile acid malabsorption [2–4]. Ulcerative colitis (UC) is a common chronic form of colitis that has a long course, which is difficult to treat [5]. The clinical manifestations of UC include abdominal pain, mucus production, diarrhea, and bloody stools [6]. The incidence and prevalence of UC are continue to increase, with North America and Europe having the highest prevalence [7]. The inflammation in UC is mainly concentrated in the colon (95%), and the terminal ileum is also affected in some patients, making it indistinguishable from Crohn's disease [8, 9]. Studies have shown that abnormal responses to environmental factors, genetic susceptibility, abnormal immune regulation, intestinal mucosal barrier, and altered intestinal microecology are important factors in the development of UC [10]. Various treatments are available for UC, including anti-inflammatory, immunosuppressive, and biological therapies; however, a large number of patients with UC do not benefit from these treatments because of their insensitivity or significant treatment-related adverse effects [11]. 5-Aminosalicylic acid, mesalazine, steroids, and immunosuppressants are the main drugs used to treat UC [9]. Studies have found that drug therapy can cause resistance and complications, and the patients had to undergo colectomy [12]. Therefore, treatment efficacy, safety, and quality of life should be considered in the future for patients with UC.

Inflammatory destruction and repair of the intestinal mucosa are key features of inflammatory bowel disease (IBD). In the gastrointestinal tract, tight junction proteins are located at the interface between the epithelial cells, which are responsible for maintaining the integrity and function of the intestinal mucosal barrier [13]. The intestinal epithelial cells play a key role in maintaining homeostasis between the microbiota and the host, producing physical barriers such as mucus layers, glycocalyxes, and cell junctions and chemical barriers that subtly isolate the symbiotic microorganisms from host immune cells to prevent unnecessary conflicts between symbiotic microbes and host immune cells, thus maintaining symbiotic relationships in the gut [14]. Bacterial or endotoxin invasion is primarily recognized by Toll-like receptor 4, a typical member of the pattern recognition receptor family, which recognizes bacterial components, such as lipopolysaccharides, and can trigger a signaling cascade leading to activation of NF-*κ*B, which triggers a series of proinflammatory immune responses through the host's attempt to destroy the invading pathogen [15, 16]. Disruption of the mucosal barrier in patients with UC can trigger bacterial and endotoxin translocation, leading to loss of innate immunity and abnormal activation of acquired immunity [17]; thus repair of the intestinal mucosal barrier is crucial in the treatment of colitis.

In recent years, a more popular theory has suggested that dysregulation of the gut immune response to microorganisms is one of the causes of onset, progression, and changes in UC [18]. The intrinsic and adaptive immunities of the host prevent the invasion of harmful bacteria and tolerate normal microbiota. However, an imbalance in the intestinal flora leads to a decrease in intestinal immune function and overstimulation of the intestinal mucosal immune response, which eventually leads to the development of IBD [19]. Gut microbiota disorders, also known as ecological dysbiosis or flora imbalance, can promote susceptibility to colitis-associated tumors by overstimulating CD8 T cells, promoting chronic inflammation and early T-cell failure and thereby reducing antitumor immunity [20]. One study found that gut microbes have an important role in brain function and behavior [21]. Vamanu et al. [22] found that the dynamic activity of the microbiota can prevent the development of degenerative diseases through food control and consumption of new active ingredients. The composition of the three major bacterial phyla in the intestinal flora of patients with IBD was found to be disturbed, with a decreased proportion of thick-walled and anaphylactic phyla and an increased proportion of anaphyla [23]. The gut microbial composition is a powerful inducer of intestinal pro-inflammatory T helper 17 cells (Th17) and regulatory T cells (Treg). Through the induction of type 3 retinoic acid-related orphan receptor *γ*t (ROR*γ*t) (+), Tregs cells and Th17 cells regulate type 2 responses and balance mucosal immune responses [24]. During UC progression, inflammation leads to a usual increase in the number of Th17 cells and a decrease in Treg that suppresses Th17 activity [25]. The intestinal flora can interact with the metabolites such as bile acids (Bas) to regulate host metabolism by regulating the microbial composition of the body, mainly through the activation of immune genes in the small intestine [26, 27]. According to the literature, BAs play an important role in intestinal immune homeostasis by regulating immune and inflammatory processes through signaling pathways, such as the farnesoid X receptor (FXR) regulatory pathway and cell surface G protein-induced signaling [28], as well as by controlling the release of immunoglobulin A antibodies from intestinal microbes [29]. One study found that short-chain fatty acids (SCFAs) such as acetate, propionate, and butyrate were able to produce bacterial species with metabolites that had positive effects on the intestinal mucosa [30]. Butyrate is the main energy source of colonocytes that has anti-inflammatory and intestinal homeostasis effects [31, 32]. Thus, the interaction between the intestinal flora, mucosal immunity, and BA metabolism plays a key role in the pathogenesis of UC.

Trans-anethole (TA), C_10_H_12_O, is the main component extracted from aniseeds, anises, and fennels. It has anti-inflammatory, antioxidant, anticancer, neuroprotective, and vascular activities [33]. TA ameliorates chronic lung inflammation in mice with chronic obstructive pulmonary disease [34]. TA also inhibits Th2 cytokines to reduce ovalbumin-induced airway inflammation [35]. However, it is unknown whether anethole has anti-inflammatory and immunomodulatory effects in colitis. Herbal monomeric compounds have been developed to treat UC. Studies have shown that baicalin attenuates the Th17/Treg ratio and reduces the ratio of thick-walled to anaphylactoid phyla and the level of endotoxin-containing anaphylactoid phyla in the stool of rats with UC and can be used as a prebiotic agent for the treatment of inflammation and intestinal ecological dysregulation associated with UC [36]. Berberine improves Treg/Th17 homeostasis in dextran sulfate sodium salt (DSS)-induced UC models, decreases the diversity of intestinal microorganisms, and interferes with the relative abundance of *Desulfovibrio*, *Eubacterium*, *and Bacteroides* [37]. This demonstrates the promising potential of the active ingredients of herbal medicines for the prevention and treatment of UC.

We hypothesized that anethole could alleviate intestinal inflammation by inhibiting the inflammatory response, restoring intestinal mucosal barrier function, modulating the immune response, restoring the intestinal flora, and altering the composition of SCFAs and BAs. Therefore, this study was conducted to investigate the mechanism of action of anethole in the pathological process of colitis by constructing a mouse model of DSS-induced colitis and to provide a reference for the development of therapeutic drugs for colitis.

## 2. Materials and Methods

### 2.1. Animal

Forty SPF, 6–8 weeks old, BALB/c mice weighing 20–25 g were obtained from Three Gorges University. All experiments were approved by the Animal Ethics Committee of Wuhan Myhalic Biotechnology Co., Ltd. (HLK-20220520-003) and performed in accordance with the National Institutes of Health Guidelines for the Care and Use of Laboratory Animals. Experimental animal use license number: SYXK (E) 2018-0104. The mice were divided into five groups of eight mice each. All mice were kept for 14 days on a normal diet, and the breeding environment was set at a temperature of 22–26°C with a relative humidity of 50%–60% and artificial light and dark illumination for 12 hr each mouse; weight changes were measured daily, and blood in the stool was observed and recorded. The mice were fed water (Table 1). DSS was purchased from Sigma (D8906). On days 8–14, the TA (Aladdin, A111314) group was gavaged with 62.5 mg/kg TA, the prednisolone group (Pre, Aladdin, P276607) was gavaged with 10 mg/kg prednisolone, and the probiotic group (Pro, OsteoForm, Q/SHKJ0003S) was gavaged with 1 × 10^9^ cfu/2 mL probiotics (once daily gavage). The mice were anesthetized with sodium pentobarbital on day 15 and sacrificed by cervical dislocation. Blood, colon, ileum, feces, and ileocecal contents were collected from the eyes of mice. The serum, feces, and ileocecal contents were stored at −80°C. Some of the colon and ileal tissues were placed in tissue fixative, and some were stored at −80°C.

### 2.2. Hematoxylin and Eosin (HE) Staining [38]

The colon was embedded, frozen at −20°C, and 3 *μ*m sections were cut, water-bath spread, and baked. Sections were stained with hematoxylin (Beyotime, Shanghai, China) for 5 min, followed by rinsing under running tap water for 3 min first and then for 20 s. Sections were then counterstained with eosin (Solarbio, Beijing, China) for 4 min, followed by cleaning with 80% and 95% ethanol (Macklin, Shanghai, China) for 40 s each. Sections were then cleared in xylene (Macklin, Shanghai, China) for 5 s and mounted with neutral balsam. After staining, the sections were observed under a microscope, and the Leica Application Suite was used to collect and analyze the relevant parts of the samples.

### 2.3. Assessment of Colonic Inflammation

Colon length was measured and photographed. Colonic inflammatory cell infiltration and colonic epithelial damage were used for scoring criteria. Scores ranged from 0 to 6 (1) few inflammatory cells with no epithelial degeneration; (2) mild inflammation with few signs of epithelial degeneration; (3) moderate inflammation with few epithelial ulcers; (4) moderate-to-severe inflammation with ulcers in 35%–50% of tissue sections; (5) moderate-to-severe inflammation with ulcers in >50% of tissue sections; (6) severe inflammation with ulcers in >75% of tissue sections) [39]. For all HE staining results, three samples from each group were blindly selected for scoring, and the order of each group was altered.

### 2.4. TUNEL Staining

The wax-embedded mouse colon tissue was immersed, freezed, and cut into 3 *μ*m sections; the sections were attached to the slides and baked at 65°C. The slides were soaked in xylene for 25 min and treated with graded alcohol 100%–75% for 3 min. Proteinase K (Solarbio, P1120) was added in 37°C environment for 15 min. TUNEL mix (ROCHE, 11684817910) was then added, followed by incubation for 60 min, POD (ROCHE, 11684817910). After transformation, DAB (Solarbio, Beijing, China) was added, slides were washed, stained with hematoxylin, and photographed with drops of neutral resin.

### 2.5. Immunohistochemical Staining (IHC) [38]

Immunohistochemistry was used to investigate the protein expression of MUC2 (1 : 200, PAB39041, Bioswamp), ZO-1 (1 : 200, PAB36669, Bioswamp), occludin (1 : 100, PAB33418, Bioswamp), TGF-1 (1 : 200, PAB39276, Bioswamp), and VEGF (1 : 100, PAB30976, Bioswamp) in the colon tissues. The colon tissues were embedded in paraffin and cut into a thickness of 5 *µ*m. Xylene transparency, ethanol dehydration, microwave high-pressure antigen repair, and goat serum blocking were performed. The polyclonal antibody was incubated for 12 hr, and the secondary antibody (MXB, Fuzhou, China) was incubated for 30 min. After DAB and hematoxylin staining, the slides were coated with a resinous mounting material and coverslips. The images were acquired using a Leica microsystem. All antibodies were purchased from Bioswamp (dilution, 1 : 200).

### 2.6. Western Blot Analysis [38]

The colon tissues were lysed in RIPA buffer (Solarbio, Beijing, China). The proteins were collected by centrifugation. A Kit (Solarbio, Beijing, China) was used to determine the concentrations. SDS–PAGE and wet transfer were used to collect proteins from the membranes (Millipore, Billerica, MA, USA). Skim milk powder blocks the receptors. Polyclonal antibodies, including anti-FGF15 (ab229630) and anti-p-NF-kB (ab76302) antibodies from Abcam (UK), anti-Ang4 antibody from Invitrogen (USA), anti-claudin-1 (PAB33156), anti-occludin (PAB33418), anti-ZO-1 (PAB36669), anti-FXR (PAB35809), anti-ASBT (PAB38035), anti-BSEP (PAB39058), anti-HMGB1(PAB36682), anti-NLRP3 (PAB37930), anti-NF-kB (PAB34738), anti-intestinal fatty acid-binding protein (I-FABP) (PAB32901), and anti-GAPDH (PAB36269) antibody from Wuhan Bioswamp (China), were incubated for 12 hr. The secondary antibody (SAB43714, Bioswamp, 1 : 20,000) was incubated for 2 hr. Images were captured using an automatic chemiluminescence analyzer. The antibody dilution ratio was 1 : 1,000.

### 2.7. ELISA

Colon tissues and serum were collected from the blood. Interleukins 10 (IL-10, MU30055), 8 (IL-8, MU30010), and 17 (IL-17, MU30074); tumor necrosis factor *α* (TNF-*α*, MU30030); interferon *γ* (IFN-*γ*, MU30038); and forkhead box P3 (FOXP3, MU30966) were detected using the ELISA kits from Bioswamp (China). RAR-related orphan receptor *γ*t (ROR*γ*t, JYM1078Mo) levels were detected using the ELISA kits from Wuhan Gene Beauty Biotechnology Co., Ltd.

### 2.8. Transmission Electron Microscopy

The colons were fixed with glutaraldehyde and osmic acid (EmCN, Beijing, China), followed by phosphate buffered solution (PBS, Solarbio, Beijing, China) wash; tissues were then dehydrated in alcohol series, embedded in acetone and epoxy resin (Emcn, Beijing, China), sectioned at a thickness of 60 nm, and stained with uranyl acetate (Mianzhu Dingtian Chemical Co., LTD, Deyang, China) and lead citrate (Emcn, Beijing, China). The resulting sections were subjected to ultrastructural examination using transmission electron microscopy.

### 2.9. Biochemical Test [38]

A Shenzhen Mindray BS-420 automatic biochemical analyzer was used to detect serum, and diamine oxidase (DAO), glutathione (GSH), malondialdehyde (MDA), myeloperoxidase (MPO), and superoxide dismutase (SOD) concentrations were used in the biochemical kit.

### 2.10. Flow Cytometry

The isolate (TBD, LDS1090) was added to the blood and centrifuged at 400 *g* for 30 min. The second layer of liquid was transferred to PBS, centrifuged at 250 *g* for 10 min, and the supernatant was aspirated to obtain mouse peripheral blood mononuclear cells (PBMC). Treg cell assay was formed by adding 100 *μ*L PBS, 2 *μ*L CD4-FITC (11-0041-81), and CD25- APC (17-0251-81), incubating at 4°C for 30 min at 400 *g*, and centrifuging for 5 min. A fixative (BD bioscience, 562574) was then added, followed by centrifuging for 5 min at 1,500 rpm after 10 min, adding PBS and 2 *μ*L of Foxp3-PE (12-5773-82), incubating for 45 min at avoidance of light, and assaying. Th17 cell assay was prepared as per the following steps: 1 × 10^6^ PBMC were taken and incubated for 6 hr with 2 *μ*L of stimulation blocking synthase (sigma, P1585), ionomycin (solarbio, I8800), and monensin sodium (solarbio, M8670); 100 *μ*L buffer and 2 *μ*L CD4-FITC was added, incubated for 30 min avoiding light, added fixative, centrifuged at 1,500 rpm for 5 min after 10 min, added PBS and 2 *μ*L of IL-17-PE (12-7177-81), incubated for 45 min avoiding light and assayed. All antibodies were obtained from eBioscience.

### 2.11. High Throughput 16S rDNA Sequencing

The DNA of the microflora was extracted, the concentration and purity of the DNA samples were checked using a Nanodrop, and the integrity of the DNA samples was checked using agarose gel electrophoresis. Specific segments of the variable region (V3–V4) were selected for PCR amplification, purification, and recovery of the amplification product using magnetic beads, fluorescence quantification of the amplification product, the fluorescence reagent Quant-it PicoGreen dsDNA Assay Kit, and a Microplate Reader (BioTek, FLx800). The Illumina TruSeq Nano DNA LT Library Prep Kit was used to prepare the sequencing libraries. Library size distribution was measured using an Agilent 2100 Bioanalyzer, and library concentration was determined by Qubit 3.0 or fluorescent quantitative PCR. The libraries were then sequenced, and the data were analyzed.

### 2.12. Gas Chromatography–Mass Spectrometry

Standards: acetic, propionic, isobutyric, butyric, isovaleric, valeric, and caproic acids were purchased from Sigma-Aldrich. Serum was added to a 2 mL centrifuge tube; 50 *μ*L of 15% phosphoric acid, 10 *μ*L of 75 *μ*g/mL internal standard (isocaproic acid) solution, and 140 *μ*L of ether were vortexed and shaken for 1 min; and the supernatant was centrifuged at 4°C for 10 min at 12,000 rpm. The supernatant was collected and analyzed using a gas chromatography–mass spectrometer (Suzhou Anyipu Precision Instrument Co., Ltd.).

Chromatographic conditions: agilent HP-INNOWAX capillary column (30 m^*∗*^ 0.25 mm ID^*∗*^ 0.25 *μ*m); split injection with a volume of 1 *μ* and split ratio of 10 : 1; and 250, 300, and 250°C temperatures for the injection port, iron source, and transmission line, respectively. The programed ramp-up temperature started at 90°C, followed by ramping up to 120°C at 10°C/min, 150°C at 5°C/min, and finally 250°C at 25°C/min for 2 min. Helium was used as the carrier gas at a flow rate of 1.0 mL/min.

Mass spectrometry conditions were as follows: electron bombardment ionization (EI) source, SIM scan mode, and electron energy of 70 eV.

### 2.13. Liquid Chromatograph Mass Spectrometer

Standards: isoLCA, NorDCA, alloLCA, LCA, 6-ketoLCA, 12-ketoLCA, 7-ketoLCA, *β*-UDCA, DCA, CDCA, UDCA, 7,12-diketoLCA, 6,7-diketoLCA, HDCA, NorCA, DHCA, *α*-MCA, UCA, *β*-MCA, CA, ACA, *β*-CA, T-*β*-MCA, GLCA, GHDCA, GCDCA, GUDCA, GDCA, LCA-3S, TCDCA, GCA, TLCA, TDCA, TCA, T-*α*-MCA, THCA, CDCA-G, TUDCA, and THDCA. The serum was added to a 2 mL EP tube, 600 *μ*L of methanol (−20°C) was added accurately, vortexed for 60 s, centrifuged at 12,000 rpm for 10 min at 4°C, and the supernatant was concentrated and dried by passing 400 *μ*L through 0.22 *μ*m filter membrane; 100 *μ*L of 30% methanol redissolved sample was taken, vortexed for 30 s, and detected using liquid chromatography (SCIEX, EXIONLC AD).

Chromatographic conditions: agilent HP-INNOWAX capillary column (30 m^*∗*^ 0.25 mm ID^*∗*^ 0.25 *μ*m); split injection with a volume of 1 *μ*L and split ratio of 10 : 1; and 250, 300, and 250°C temperatures for the injection port, iron source, and transmission line, respectively. The programed ramp-up temperature started at 90°C, followed by ramping up to 120°C at 10°C/min, 150°C at 5°C/min, and finally 250°C at 25°C/min for 2 min. Helium was used as the carrier gas at a flow rate of 1.0 mL/min.

Mass spectrometry conditions were as follows: electron bombardment ionization (EI) source, SIM scan mode, and electron energy of 70 eV.

### 2.14. Statistical Analyses

All data are presented as means ± SD. The SPSS v23.0 software was used for data analysis. One-way ANOVA was used to compare data between multiple groups, and *t*-test was used to compare two groups. *P* < 0.05 was considered statistically significant.

## 3. Results

### 3.1. DSS-Induced Colitis in BALB/c Mice

**Figure 1:**
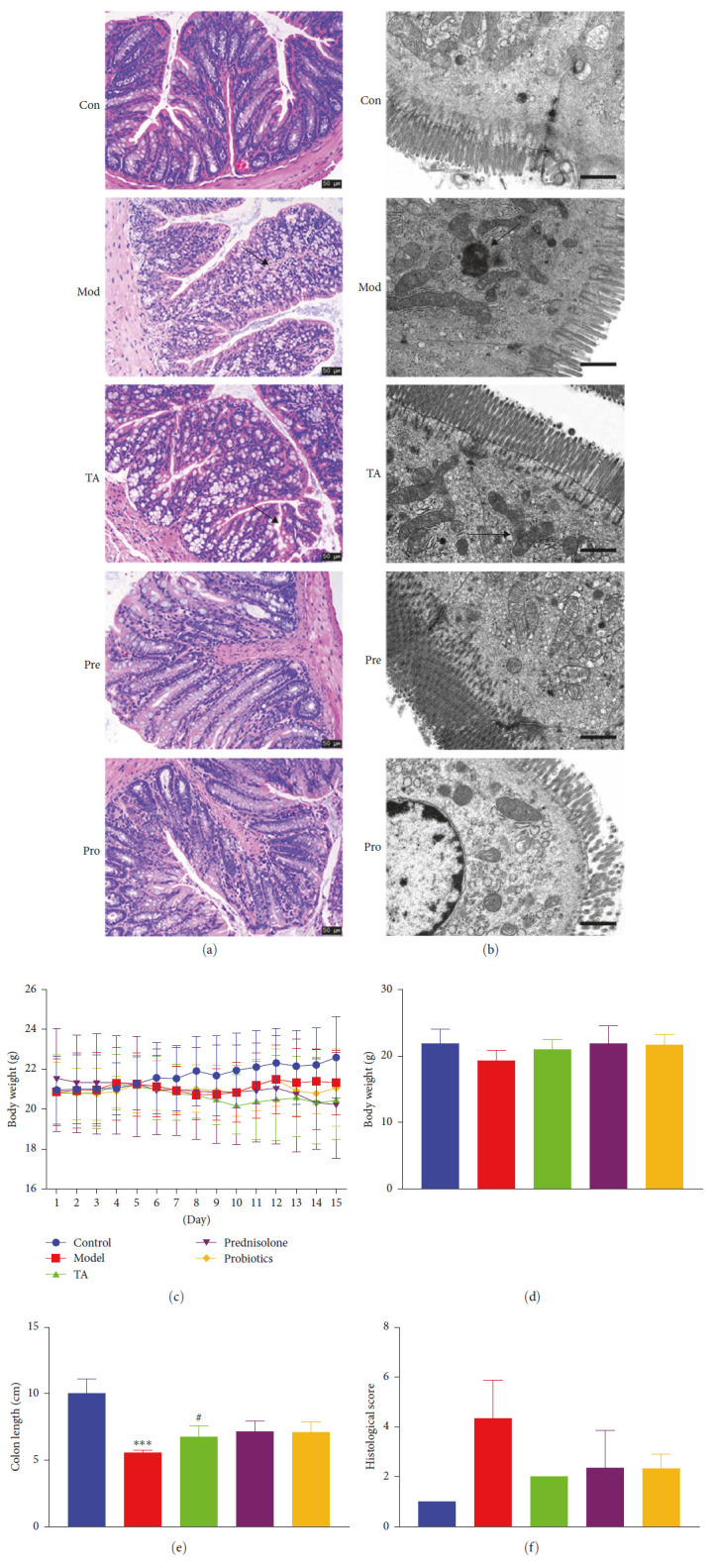
DSS-induced colitis in BALB/c mice: (a) HE staining of the colon tissue, Scala bar = 50 *μ*m; (b) ultrastructure of the colon was observed using transmission electron microscopy, Scala bar = 1 *μ*m; (c) body weight changes in mice with UC; (d) body weight of mice with UC on day 15; (e) colon length of mice with UC; (f) histological score.

HE staining at week 15 showed that DSS feeding for 11 weeks caused colonic lesions in BALB/c mice. Compared with the control group, the colon crypt in the model group was swollen and destroyed; lymphocytic infiltration, diffuse mucosal congestion, edema, granular mucosal surface, increased mucosal fragility, and ulceration were noticed. The crypt surface was uneven, the crypt gap was enlarged, and the epithelium was damaged. After TA treatment, the mucosal layer remained intact, the crypt gap was reduced, and a U-shaped crypt was visible (Figure 1(a)–1(f)). TEM revealed damaged ileal intestinal mucosa, reduced microvilli, and incomplete mitochondria in the model group (Figure 1(b)). The body weight of mice gradually decreased after DSS feeding, and the body weights of mice in the TA, pre, and pro groups did not decrease at the beginning of treatment (Figures 1(c) and 1(d)). In addition, the colons of the mice in the model group were significantly shortened, and the length of the colons in the TA group was increased compared to that in the model group (Figure S1).

### 3.2. Effect of TA on the Intestinal Barrier in Ulcerative Colitis

**Figure 2:**
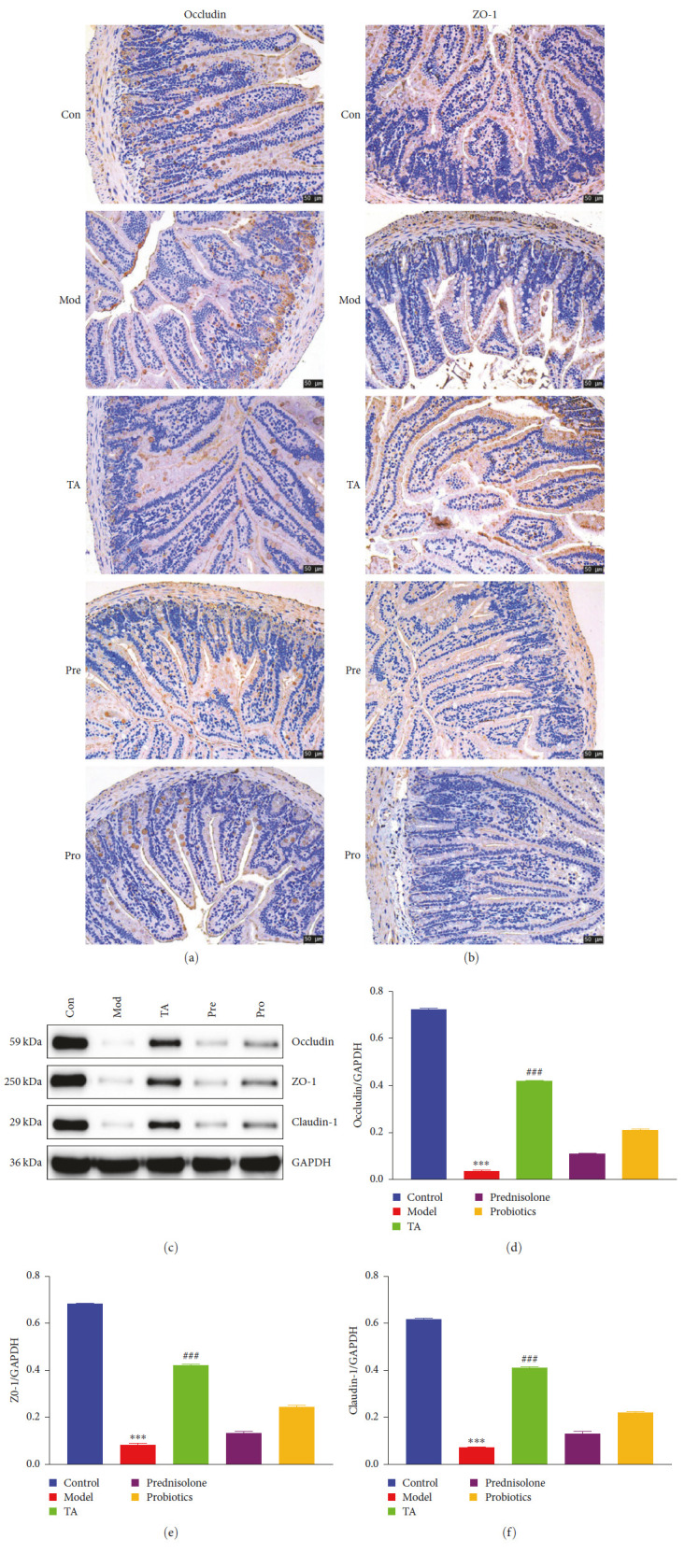
Western blot and IHC detected the protein expression levels of claudin-1, occludin, and ZO-1. Scala bar = 50 *μ*m.

**Figure 3:**
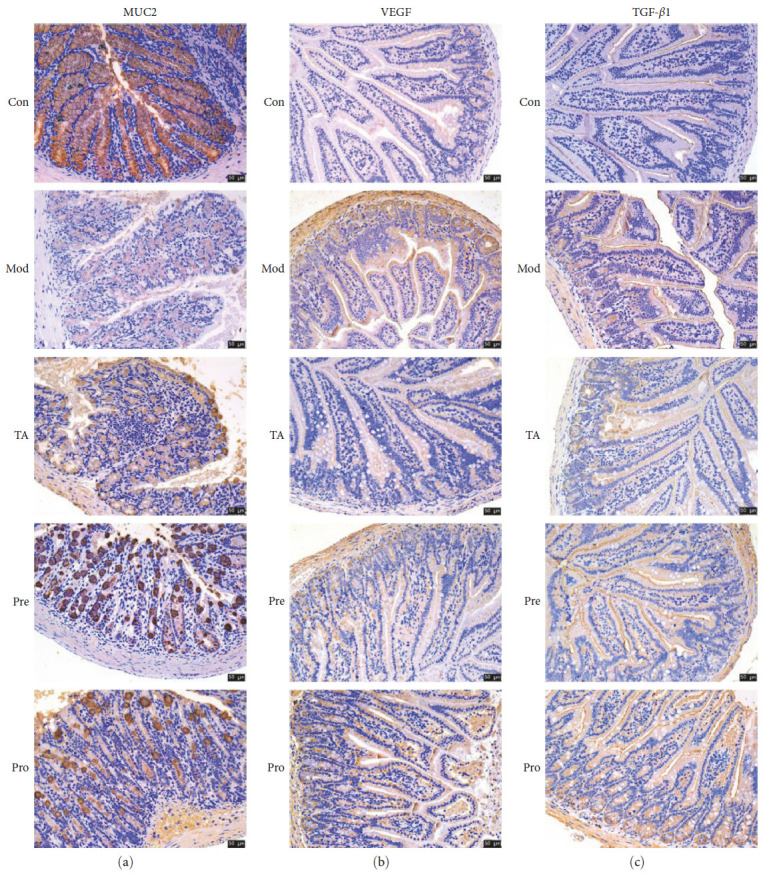
IHC detected the protein expression levels of MUC2, VEGF, and TGF-*β*1. Scala bar = 50 *μ*m.

The expression levels of claudin-1, occludin, and ZO-1 in the colons of mice in the model group were significantly lower than those in the colons of mice in the control group (*P* < 0.05). The expression levels of claudin-1, occludin, and ZO-1 were significantly higher in the TA group than those in the model group (*P* < 0.05). This suggests that intestinal barrier function is impaired in UC, and TA plays a role in regulating impaired intestinal barrier-related protein repair (Figure 2(a)–2(f)). In addition, MUC2 protein expression levels were decreased, and VEGF and TGF-*β*1 protein expression levels were increased in the colon of the model group. Compared to the model group, MUC2 protein expression levels increased, and VEGF and TGF-*β*1 protein expression levels decreased in the TA group (Figure 3(a)–3(c)).

### 3.3. TA Alleviates Apoptosis in Ulcerative Colitis

**Figure 4:**
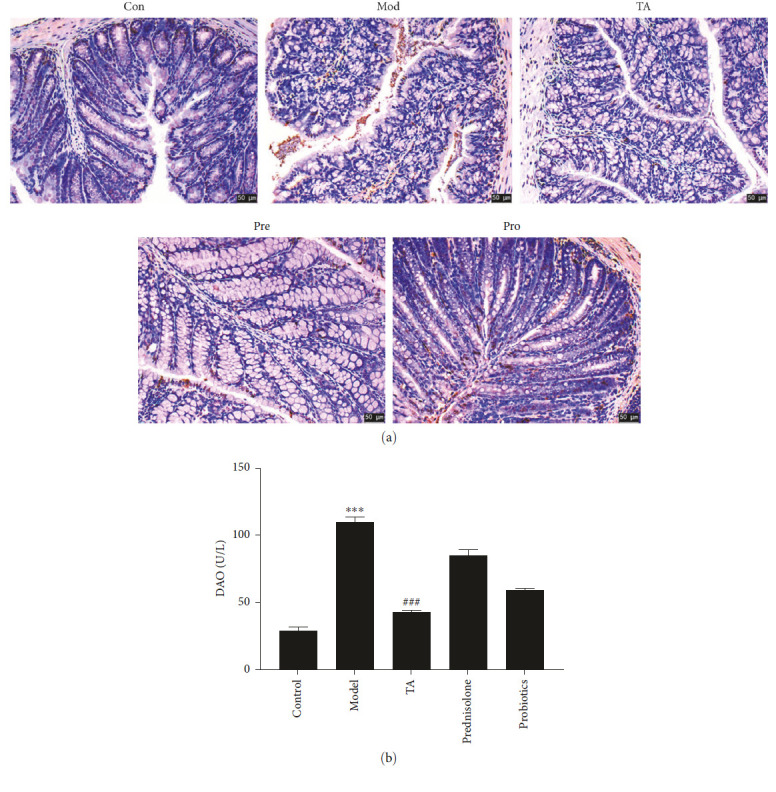
Trans-anethole alleviates apoptosis in ulcerative colitis: (a) TUNEL detected the apoptosis. Scala bar = 50 *μ*m; (b) ELISA detected DAO content.

Compared to the control group, apoptotic cells were found in the damaged colon at this time point, and there were no significant numbers of apoptotic cells in the TA group compared to the model group (Figure 4(a)). Moreover, the amount of DAO was significantly higher in the model group (*P* < 0.05) and lower in the TA group (*P* < 0.05) than that in the control group (Figure 4(b)). Impaired colonic tissue and intestinal barrier function were associated with apoptosis of the colon cells.

### 3.4. TA Relieves Inflammation in Ulcerative Colitis

**Figure 5:**
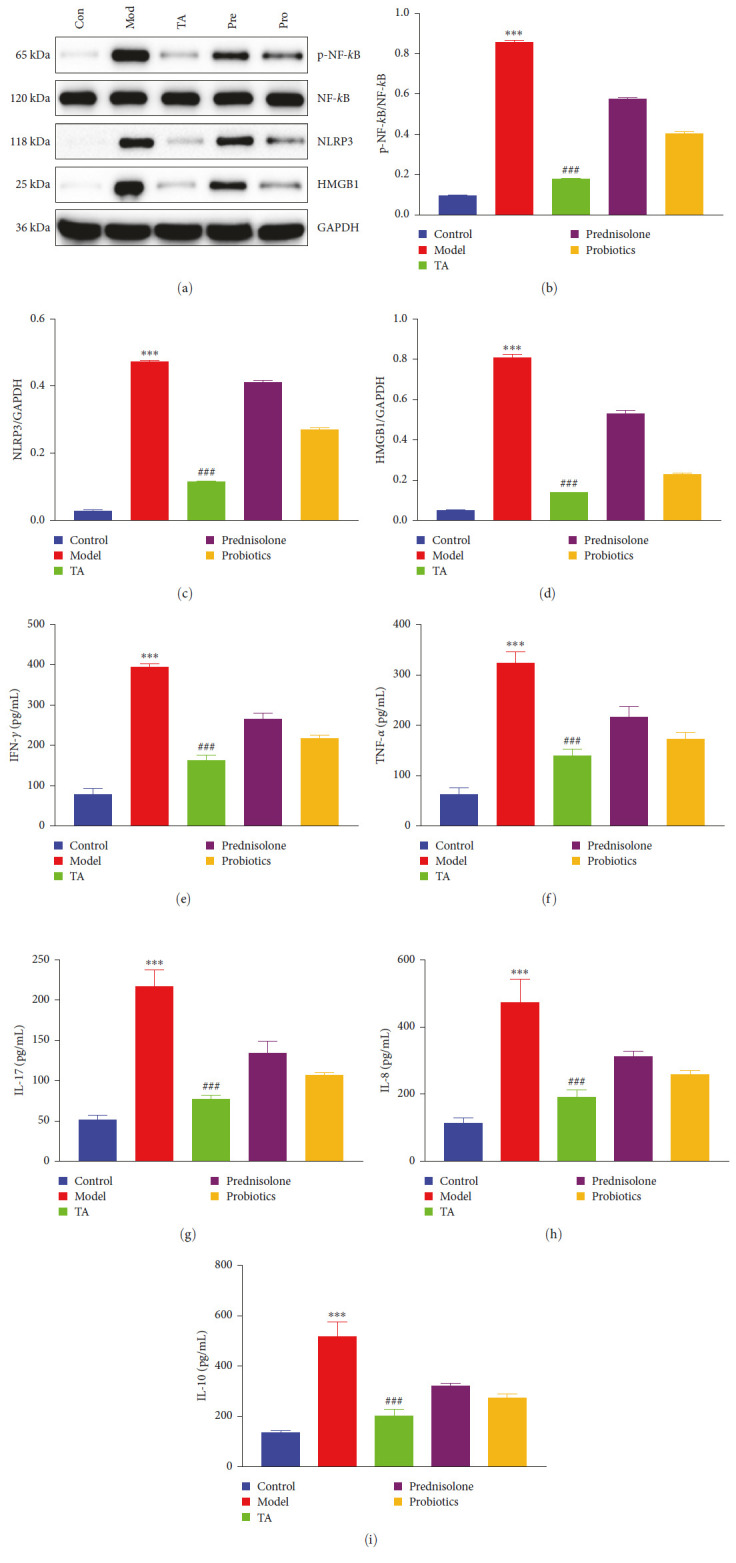
Trans-anethole relieves inflammation in ulcerative colitis: (a–d) western blot detected the NF-kB pathway; (e–i) ELISA detected IFN-*γ*, TNF-*α*, IL-17, IL-8, and IL-10 content in the colon.

Apoptosis in the colonic tissue may be the reason for this. By detecting the levels of inflammatory signaling pathways and inflammatory factors in the colonic tissues, it was found that p-NF-kB inflammatory signaling pathway was activated in the model group. The expression levels of HMGB1, NLRP3, NF-kB, and p-NF-kB proteins were significantly lower in the TA group compared with the model group (*P* < 0.05, Figure 5(a)–5(c)). Similarly, the levels of IL-10, IL-8, IL-17, and TNF-*α* were significantly higher in the model group than those in the control group (*P* < 0.05), and the levels of IL-10, IL-8, IL-17, and TNF-*α* were significantly lower in the TA group than those in the model group (*P* < 0.05; Figure 5(f)–5(i)).

### 3.5. TA Alleviates Oxidative Stress in Mice with Ulcerative Colitis

**Figure 6:**
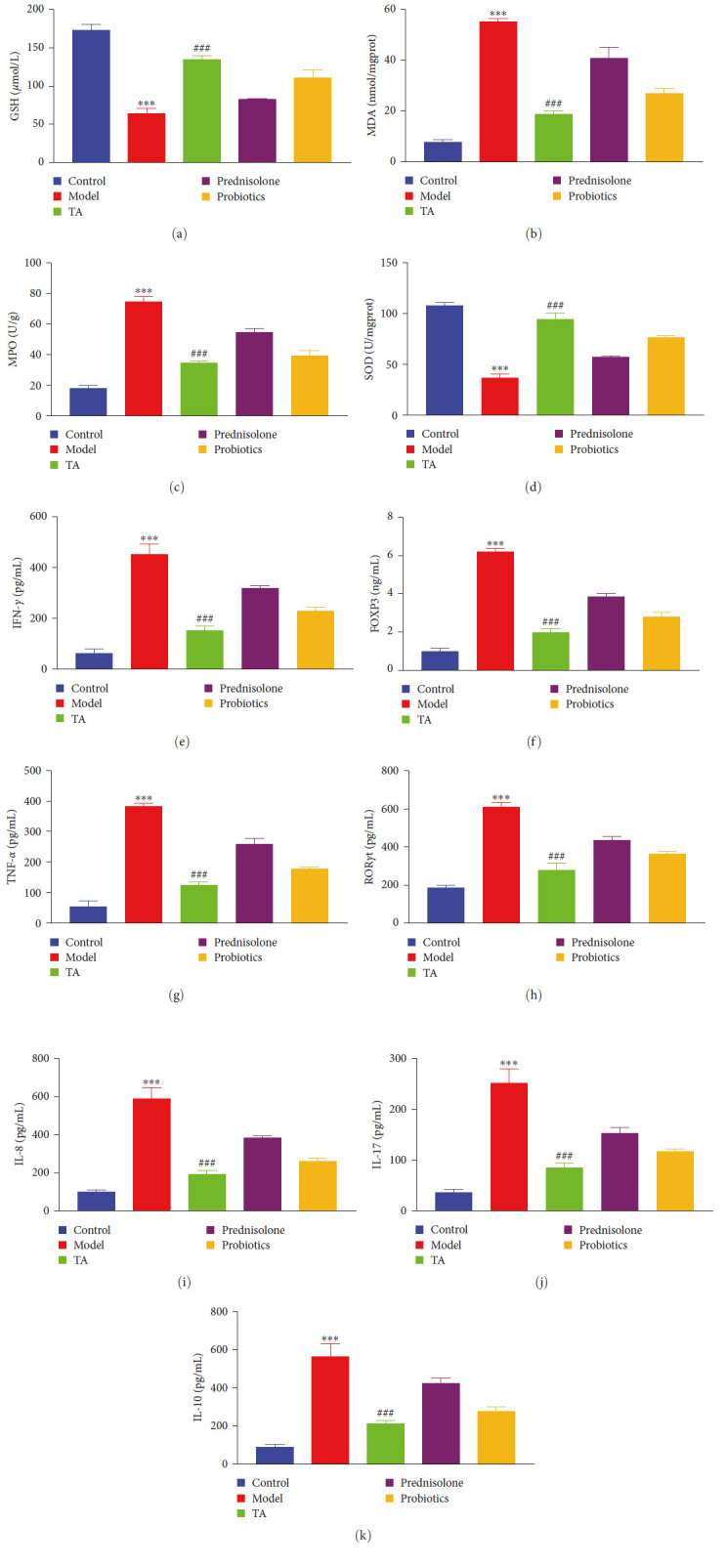
Trans-anethole alleviates oxidative stress in mice with ulcerative colitis: (a–d) biochemical tests GSH, MDA, MPO, and SOD content in serum; (e–k) ELISA detected IFN-*γ*, FOXP3, TNF-*α*, ROR*γ*t, IL-8, IL-17, and IL-10 content in serum.

Studies have shown that mice with UC exhibit severe inflammation, which leads to colonic tissue damage and triggering of apoptosis [40]. The colonic barrier (outside of the central location in the GI tract) function is also impaired; therefore, we speculate that the location of the GI damage caused by UC may not always be the colonic site. The levels of oxidative stress and inflammatory factors in the serum of mice were found to be significantly lower in GSH and SOD (*P* < 0.05) and higher in MDA, MPO, IFN-*γ*, FOXP3, IL-10, IL-8, IL-17, TNF-*α*, and ROR*γ*t (*P* < 0.05) compared with the control group. Compared with the model group, the contents of GSH and SOD were significantly increased (*P* < 0.05), and those of MDA, MPO, IFN-*γ*, FOXP3, IL-10, IL-8, IL-17, TNF-*α*, and ROR*γ*t were significantly decreased (*P* < 0.05; Figure 6) in the TA group. As a result, oxidative stress is imbalanced, and inflammation spreads in the mice with UC.

### 3.6. TA Regulates the Intestinal Flora in Mice with Ulcerative Colitis

**Figure 7:**
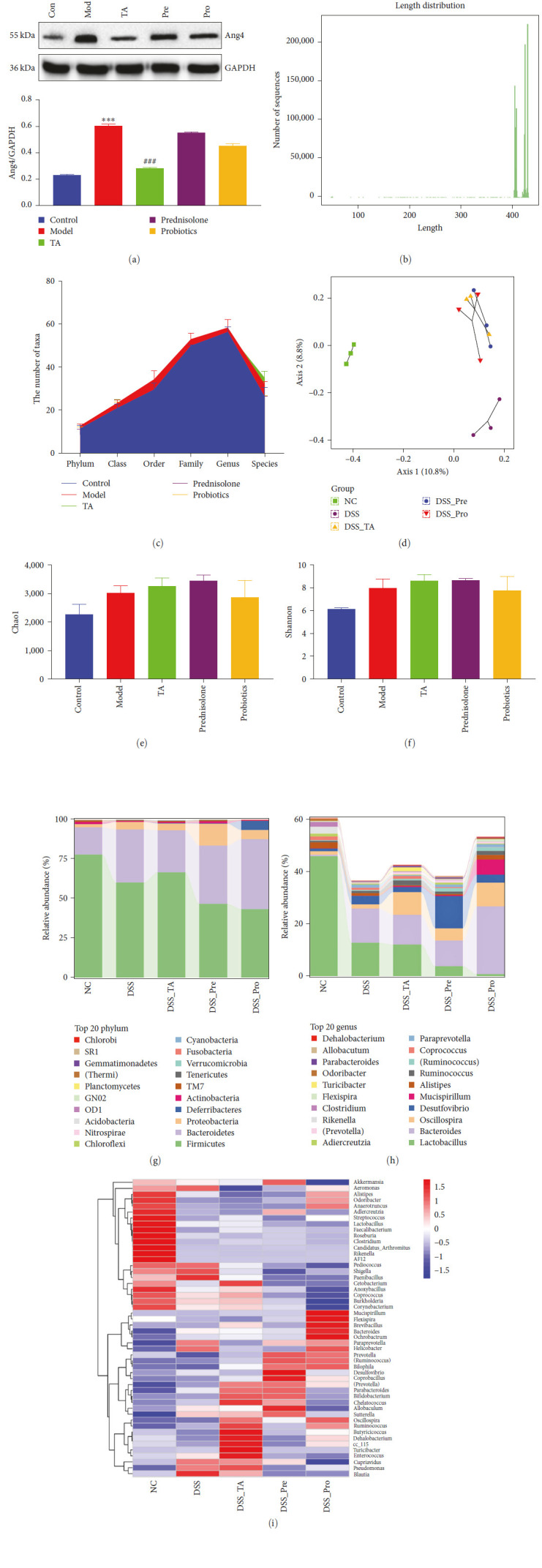
Trans-anethole regulates the intestinal flora in mice with ulcerative colitis: (a) western blot detected the protein expression levels of Ang4; (b) the sequence lengths of flora in feces and intestinal contents; (c) distribution of the number of Taxa in each group; (d) sample two-dimensional sorting chart for PCoA analysis; (e, f) microbial diversity index of the microbial community; (g) phylum abundance; (h) genus abundance; (i) heatmap of taxa clustered.

Interestingly, the Ang4 protein was found to be differentially expressed in the colonic tissues. Ang4 protein expression was significantly higher in the model group than that in the control group (*P* < 0.05) and significantly lower in the TA group than that in the model group (*P* < 0.05, Figure 7(a)). Activation of Ang4 phenotypes increases its bacteriostatic effect in the colon. Therefore, fecal samples and fresh intestinal contents from each group of mice were collected and analyzed for changes in the intestinal flora using 16S rDNA high-throughput sequencing. The sequence lengths of flora in feces and intestinal contents were concentrated at 404–431 bp, with the most abundant sequence length being 430 bp (Figure 7(b)). In the Greengenes database, the classify-sklearn algorithm of QIIME2 was used to obtain annotation information of all sequences into kingdoms, phyla, classes, orders, families, genera, and species. The resolution of annotations was higher in the model and TA groups than that in the control group. The specific composition of the microbial communities in the five groups at each taxonomic level was obtained by counting the ASV/OTU tables after drawing the level. It could be found that the number of taxonomic units was consistently highest in the model group except for the species class (Figure 7(c)). We conducted alpha and beta diversity analyses (Figures 7(e) and 7(f)) and found no significant differences in the Chao1 and Shannon indices between the groups. However, compared to the control group, the model group samples were well separated. Compared to the model group, the TA group achieved better separation. This result indicates that there was a significant difference in colony composition between the TA and model groups. *Firmicutes* and *Bacteroidetes* were the two most abundant phyla, accounting for over 80% of the total phyla level. Compared with the control group, the abundance of *Firmicutes* in the model group decreased, whereas the abundance of *Bacteroides* and *Proteobacteria* increased. Compared with the model group, the abundance of *Firmicutes* in the TA group increased, the abundance of *Bacteroidetes* decreased, and the abundance of *Proteobacteria* remained unchanged. This indicates that TA can effectively improve the imbalance of the gut microbiota in mice with colitis (Figure 7(g)). At the genus level, the control group had the highest relative abundance of *Lactobacillus*, which was the dominant bacterium. Compared to the control group, the abundances of *Lactobacillus*, *Alistipes*, *Coprococcus*, *Clostridium*, *Rikenella*, and other bacteria in the model group were reduced, whereas the abundances of *Bacteroides*, *Desulfovibrio*, and other bacteria were increased. Compared to the model group, there was no significant change in *Lactobacillus* in the TA group, but there was an increase in *Spirillum*, whereas *Bacteroides*, *Desulfovibrio Vibrio*, and other bacteria were significantly reduced. In addition, the combined strain heat map revealed that *Paraprevotella* and *Helicobacter* were abnormally increased in the model group and decreased in the TA and pro groups (Figure 7(i)), which is similar to a previous analysis of the genus *Chlorobi Phylum Oscillospira*.

### 3.7. TA Modulates the Immune Response in Mice with Ulcerative Colitis

**Figure 8:**
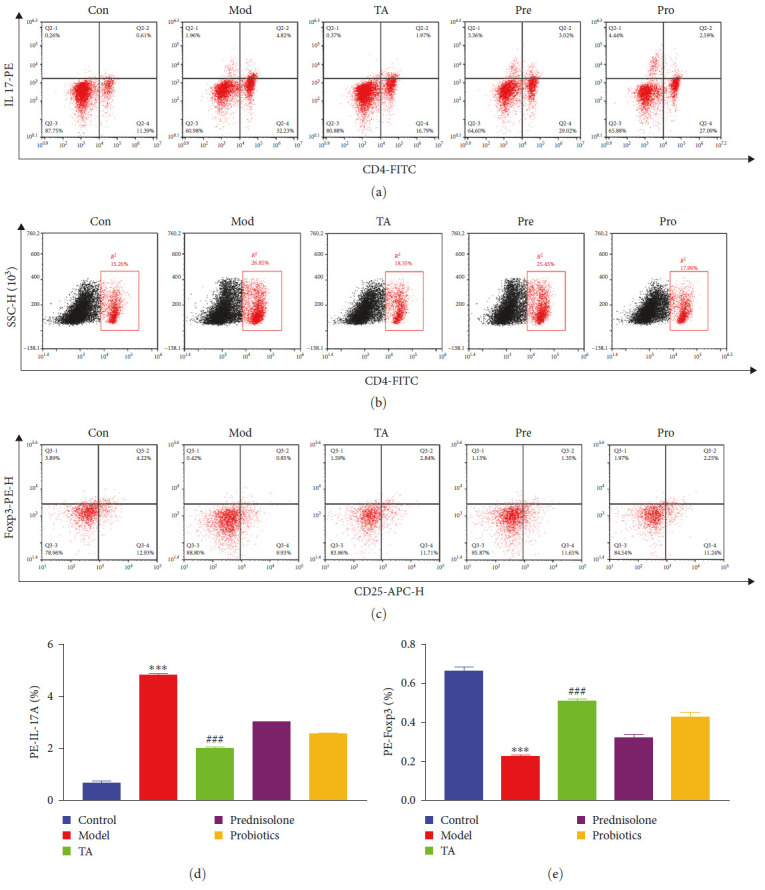
Trans-anethole modulates the immune response in mice with ulcerative colitis: (a, d) IL-17 content in CD4+ cell; (b) CD4+ cell content; (c, e) Foxp3 and CD25 content in CD4+ cell.

In mice with PBMC, an increase in Th17 cells and a decrease in Treg cells were observed in the model group compared to those in the control group. UC induced the differentiation of monocytes into Th17 cells, and TA treatment reversed this trend (Figure 8).

### 3.8. TA Regulates Fatty Acid Homeostasis in Mice with Ulcerative Colitis

**Figure 9:**
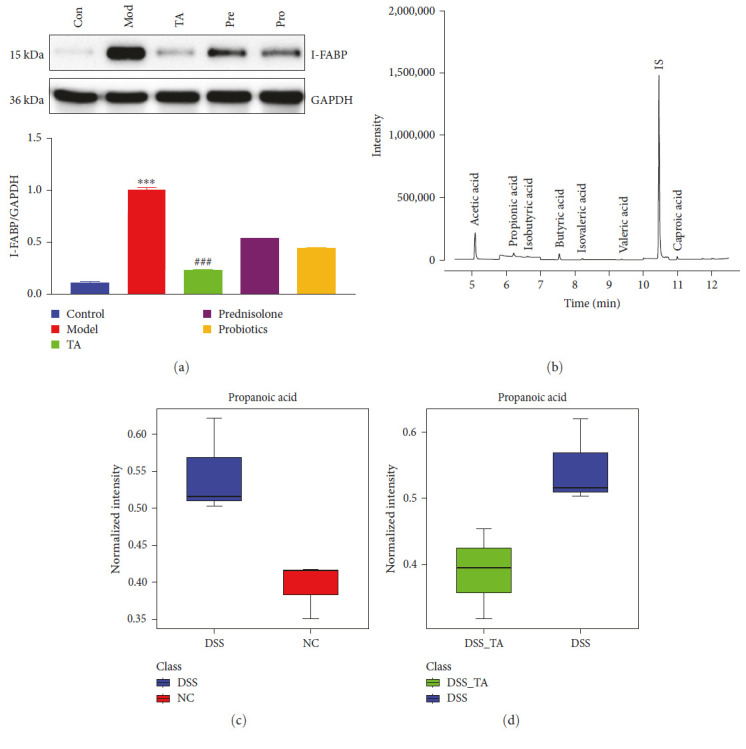
Trans-anethole regulates fatty acid homeostasis in mice with ulcerative colitis: (a) western blot detected the protein expression levels of I-FABP; (b) the total ion chromatograms of serum by GC–MS; (c) normalized intensity of propanoic acid in the Con and Mod groups; (d) normalized intensity of propanoic acid in the TA and Mod groups.

Owing to the structural incompleteness of the mitochondria in the colon, damage to the digestive tract leads to changes in energy production, as can be seen by the changes in body weight of the mice, which appear to be reduced under conditions of a normal diet, suggesting to consider energy deficiency. We found that I-FABP protein was highly expressed in the model group and that TA reduced its expression (Figure 9(a)). SCFAs and acetic, propionic, isobutyric, butyric, isovaleric, valeric, and caproic acids were detected in the serum, and butyric acid was the most abundant (Figure 9(b)). Propionic acid was elevated in the model group compared to the control group (Figure 9(c)). Propionic acid levels were lower in the TA group than those in the model group (Figure 9(d)).

### 3.9. TA Regulates Bile Homeostasis in Mice with Ulcerative Colitis

**Figure 10:**
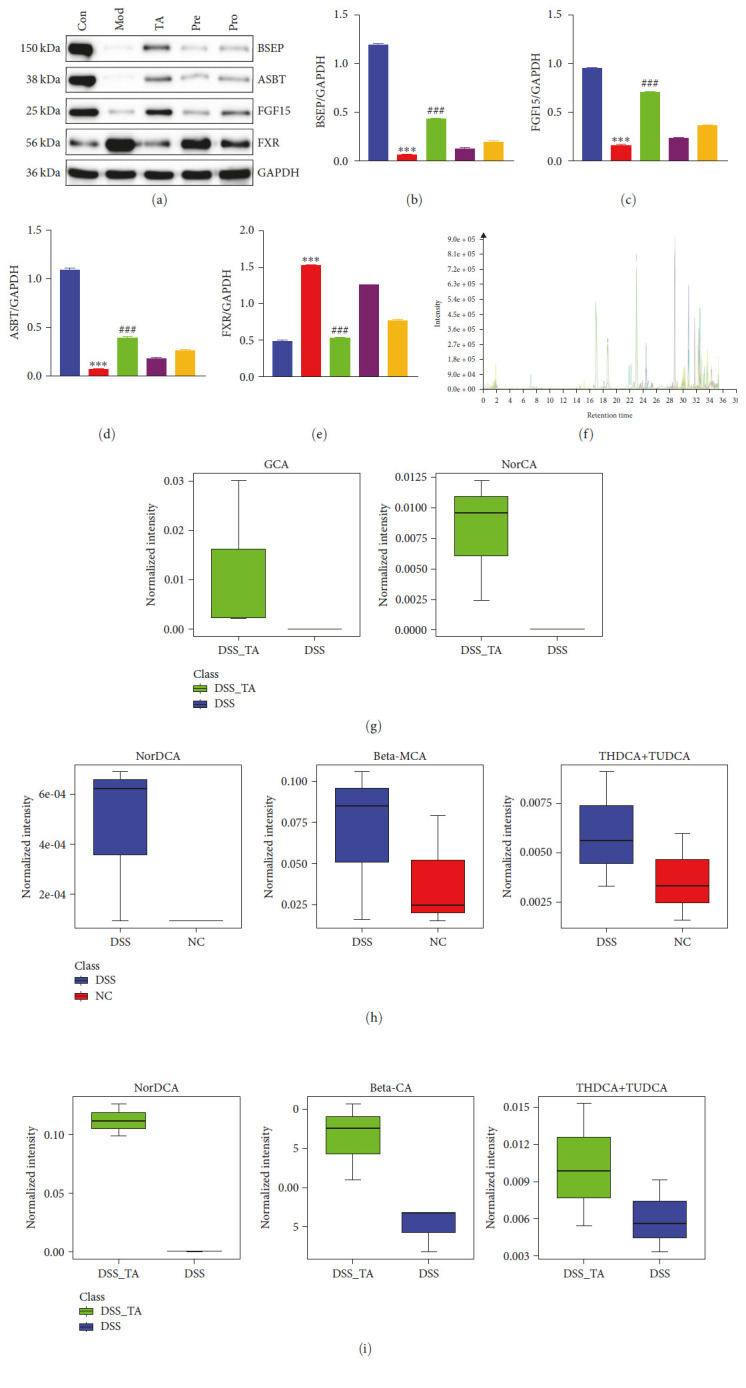
Trans-anethole regulates bile homeostasis in mice with ulcerative colitis: (a–e) western blot detected the protein expression levels of FGF15, ASBT, and BSEP in the colon; (f) the total ion chromatograms of serum by LC–MS; (g) normalized intensity of GCA and NorCA in the TA and Mod groups; (h) normalized intensity of Beta-MCA, NorDCA, and THDCA + TUDCA in the Con and Mod groups; (i) normalized intensity of Beta-MCA, NorDCA, and THDCA + TUDCA in the TA and Mod groups.

As observed in the ileum, the expression levels of FGF15, ASBT, and BSEP proteins were significantly lower in the model group, whereas the expression levels of FXR proteins were significantly higher in the control group (*P* < 0.05). Moreover, the expression levels of FGF15, ASBT, and BSEP in the TA group were significantly higher than those in the model group, and the expression levels of FXR protein were significantly lower than those in the model group (*P* < 0.05.  Figure 10(a)–10(e)). We detected the metabolic profile of Bas in the serum by LC–MS and found that bile acid metabolism was abnormal in mice with UC; NorCA and GCA were found for the first time in the TA group compared to the control and model groups (Figures 10(f) and 10(g)). Beta-MCA, NorDCA, and THDCA + TUDCA levels were elevated in the model group compared to those in the control group (Figure 10(h)). Compared to the model group, the beta-MCA, NorDCA, and THDCA + TUDCA levels were decreased in the TA group (Figure 10(i)).

## 4. Discussion

UC is an idiopathic inflammatory condition of the colon and the most common IBD worldwide. The pathology is characterized by increased fragility of the colonic wall, superficial erosion, and bleeding from the colonic wall. Inflammation occurs in the colonic mucosa and submucosa. DSS induces UC in mice. In the present study, lymphocyte infiltration, diffuse mucosal congestion and edema, a granular mucosal surface, increased mucosal fragility, and ulceration were observed in the colons of mice after DSS solution feeding. The expression level of intestinal barrier function proteins is reduced when the content of inflammatory factors in colonic cells increases, leading to the apoptosis of colonic cells. In addition, UC leads to increased levels of oxidative stress in mice, and abnormal levels of several factors have been observed in the serum. Alterations in the composition of the intestinal microbiota and mucosal immunodeficiency may contribute to UC [41]. Therefore, we speculated that disruption of the intestinal barrier and the appearance of inflammation may be associated with alterations in the intestinal microbiota and immunodeficiency. Detection of DNA sequences in the intestinal contents and feces revealed a large number of different sequences between the model and control groups. Differences in Ang4 protein expression were also observed in the colon. Second, a Th17/Treg imbalance was found by detecting the Th17 and Treg immune cell levels. A large amount of Th17-produced IL-17 was present in the model group, and similarly, abnormally high expression of Foxp3 was observed. However, the imbalance of immune cells could not be the causative factor, and the dysbiosis of the intestinal flora was directly related to the colonic tissue. Therefore, the cause of these results might also start from the alteration of the intestinal flora.

Drugs for the treatment of UC are continuously being developed and utilized. In recent years, various novel therapeutic approaches using prebiotics, probiotics, commensal bacteria, and fecal microbial transplants as complementary and alternative medicines have improved the condition of patients with IBD [42]. Probiotics can prevent human intestinal diseases; modulate the host immune response and intestinal mucosa by acting on antigens and altering the composition of the intestinal microbiota [43–45]; and repair the damaged intestinal mucosal barrier in patients with UC during disease progression [46] by inhibiting HMGB1-mediated intestinal barrier dysfunction [47], modulating immunomodulatory activity to influence cytokine expression, and maintaining the intestinal permeability by reducing inflammation and protecting intestinal barrier integrity [48]. Probiotics such as *Bifidobacterium fragilis* can improve the mucosal immune system in UC by interacting with the intestinal Treg and inducing IL-10 production [25]. Mucin Muc2 is the major glycoprotein in the colonic mucus, and MUC2 deficiency can lead to impaired epithelial barrier function, intestinal flora imbalance, and spontaneous colitis, and studies have demonstrated that the probiotic mixture VSL#3 reduces colonic inflammation and improves intestinal barrier function in Muc2 mucin deficient mice [49]. Thus, the intestinal flora can regulate intestinal barrier function, inflammation, and immune responses in mice with UC.

TA regulates intestinal barrier function, inflammation, and the immune response [50–52]. In this study, 16S rDNA high-throughput sequencing was used to analyze the intestinal flora of mice. The results showed that the abundance of *Firmicutes* in the DSS-induced colitis mouse model was significantly reduced, and the abundance of *Bacteroides* and *Denaturia* was significantly increased. After TA intervention, the imbalance in the intestinal flora significantly improved, the abundance of *Firmicutes* significantly increased, and the abundance of *Bacteroides* significantly reduced. Similar to previous studies, kaempferol reshaped the gut microbiota by increasing the ratio of *Firmicutes* to *Bacteroidetes* [53]. Zhang et al. [54] found that fecal microbiota transplantation increased the relative abundance of *Firmicutes*, reduced the abundance of *Bacteroidetes*, and restored the intestinal microbiota to normal levels. The study found that Shenlingbaizhusan increases the relative abundance of SCFA-producing bacteria at the genus level, including *Oscillospira* [55]. In this study, the abundance of *Osmospirillum* spp. was increased after anisole intervention. Therefore, TA modulates intestinal flora in mice with UC and alleviates UC. By comparing the effects of TA and probiotics, we found that both TA and probiotics increased the expression levels of claudin-1, occludin, and ZO-1 proteins in the colon, but the effect in the TA group was stronger than that in the probiotic group. By comparing the results of HE and IHC staining, it was found that the TA group had a better effect on colonic mucosa repair than the probiotic group. In addition, the protein expression levels of inflammatory signaling pathways in the TA group were lower than those in the probiotic group compared to the model group. Finally, changes in immune cell content in the colonic group showed the same advantages. Ang4 protein expression in the colonic tissue was abnormally increased in the model group and significantly decreased in the TA group compared to that in the model group. Ang4 produced by mouse Paneth cells is secreted into the intestinal lumen and exhibits bactericidal activity against intestinal microorganisms induced by *Bacillus mimicus* [56]. Thangamani et al. [57] found that taurocholic acid, a bile acid, inhibited the mRNA expression level of ang4 and regulated *Candida albicans* in the gastrointestinal tract. Combined with the LC–MS results, the percentage of secondary Bas was higher in the model group than that in the control and TA groups. This result suggests that the increased flora in the colon of mice with UC is also involved in BA metabolism. Jia et al. [58] indicated that FGF15 plays an important role in energy metabolism, including BA homeostasis, glucose metabolism, and protein synthesis. FXR is a nuclear receptor expressed mainly in intestinal tissues that regulate BA, lipid, and glucose homeostasis [59]. ASBT acts as a transporter protein responsible for ileal BA reabsorption [60]. Impaired BSEP function may lead to cholestasis in humans [61]. One study found that ASBT expression was reduced in murine, canine, and rabbit models of intestinal inflammation [62]. Zhao et al. [63] found that a high-fat diet promotes DSS-induced UC by downregulating the FXR expression through the TGFB pathway. In the present study, we observed abnormally high FXR protein expression in mice with UC, and the protein expression levels of FGF15, ASBT, and BSEP were lower than those in the control group. After TA treatment, the expression levels of FXR protein were significantly reduced, and those of FGF15, ASBT, and BSEP were significantly increased; the increase was higher than that in the probiotic group.

Laffin et al. [64] found that fatty acids modulate colitis susceptibility. Chen et al. [65] found that dandelion extract modulated fatty acid degradation and impaired microbial metabolism to ameliorate dextran sodium sulfate-induced colitis. SCFAs, anaerobic fermentation byproducts of undigested carbohydrates in the colon, were found to be an important energy source for the colonic epithelial cells [66]. In the present study, the acetate, propionate, and butyrate levels were significantly lower in the model group than those in the control group. In addition, we found that I-FABP was abnormally highly expressed in the model group, suggesting that fatty acids are not properly metabolized in the colon of mice with UC, which leads to fatty acid aggregation. Fatty acids have inherent energy-supplying properties, and impaired fatty acid metabolism in the colon of mice with UC not only leads to a reduced energy supply to the organism but also affects the mucosal barrier function. In contrast, TA reduced the fatty acid content in the colon in this study, thus alleviating the subsequent chain reaction caused by abnormal fatty acid metabolism.

In conclusion, this study demonstrated that TA can reshape the intestinal flora and reduce the production of fatty acids and BAs in DSS-induced UC and that the imbalance between inflammation and immunity in the colon of mice with UC is not only regulated by the intestinal flora but also by the reduced production of fatty acids and BAs. TA not only alleviated the impaired intestinal barrier function in mice with UC but also provided a superimposed effect on the restoration of colonic barrier function by reducing the synthesis of BAs; the same superimposed effect was also observed in Th17/Treg homeostasis.

## Figures and Tables

**Table 1:** BALB/c mice fed condition.

Group	1–7 days	8–11 days	12–14 days
Control (Con)	Water	Water	Water
Model (Mod)	5% DSS	3.5% DSS	Water
TA	5% DSS	3.5% DSS	Water
Pre	5% DSS	3.5% DSS	Water
Pro	5% DSS	3.5% DSS	Water

DSS, dextran sulfate sodium salt.

## Data Availability

Data used to support the findings of this study are available from the corresponding author upon request.

## Supplementary Materials

Supplementary Materials Figure S1: colon length.
