# Supplementary figures and images for: Trans-Anethole Alleviates DSS-Induced Ulcerative Colitis by Remodeling the Intestinal Flora to Regulate Immunity and Bile Acid Metabolism
Source: Mediators Inflamm. 2023 Sep 21;2023:4188510. doi: 10.1155/2023/4188510 (PMC10539094; doi:10.1155/2023/4188510)

Supplementary Figure 1
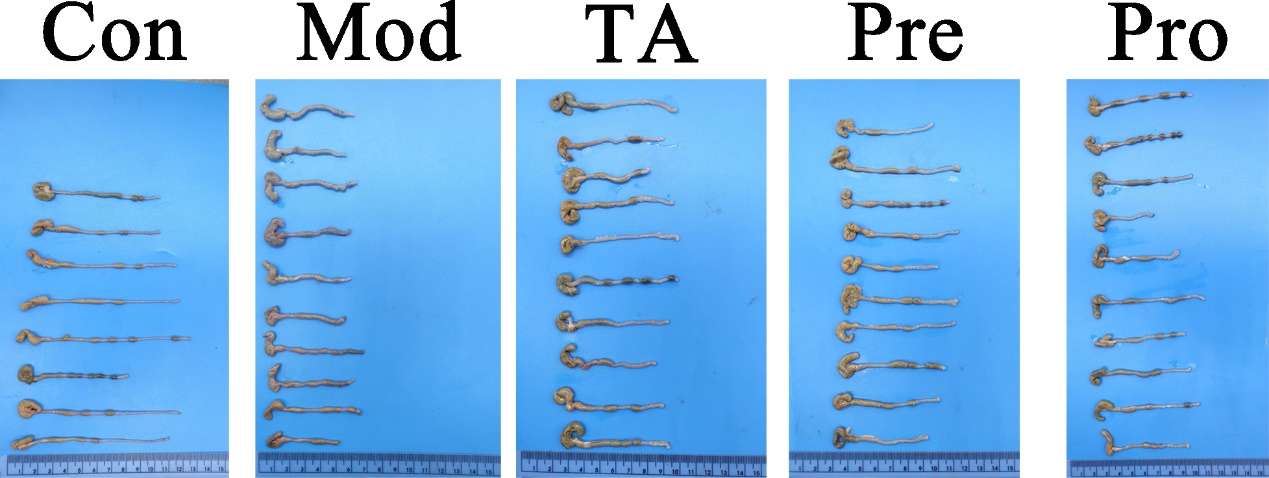


Supplementary Figure 1. Colon length.

Supplement: Supplementary Materials — Figure S1: colon length. [file 4188510.f1.docx]
